# A Novel Statistic for Genome-Wide Interaction Analysis

**DOI:** 10.1371/journal.pgen.1001131

**Published:** 2010-09-23

**Authors:** Xuesen Wu, Hua Dong, Li Luo, Yun Zhu, Gang Peng, John D. Reveille, Momiao Xiong

**Affiliations:** 1 Department of Epidemiology and Statistics, Bengbu Medical College at Bengbu, Anhui, China; 2 Laboratory of Theoretical Systems Biology and Center for Evolutionary Biology, State Key Laboratory of Genetic Engineering, School of Life Science and Institute for Biomedical Sciences, Fudan University, Shanghai, China; 3 Human Genetics Center, University of Texas School of Public Health, Houston, Texas, United States of America; 4 Division of Rheumatology, Medical School, University of Texas Health Science Center at Houston, Houston, Texas, United States of America; University of California San Diego and The Scripps Research Institute, United States of America

## Abstract

Although great progress in genome-wide association studies (GWAS) has been made, the significant SNP associations identified by GWAS account for only a few percent of the genetic variance, leading many to question where and how we can find the missing heritability. There is increasing interest in genome-wide interaction analysis as a possible source of finding heritability unexplained by current GWAS. However, the existing statistics for testing interaction have low power for genome-wide interaction analysis. To meet challenges raised by genome-wide interactional analysis, we have developed a novel statistic for testing interaction between two loci (either linked or unlinked). The null distribution and the type I error rates of the new statistic for testing interaction are validated using simulations. Extensive power studies show that the developed statistic has much higher power to detect interaction than classical logistic regression. The results identified 44 and 211 pairs of SNPs showing significant evidence of interactions with FDR<0.001 and 0.001<FDR<0.003, respectively, which were seen in two independent studies of psoriasis. These included five interacting pairs of SNPs in genes LST1/NCR3, CXCR5/BCL9L, and GLS2, some of which were located in the target sites of miR-324-3p, miR-433, and miR-382, as well as 15 pairs of interacting SNPs that had nonsynonymous substitutions. Our results demonstrated that genome-wide interaction analysis is a valuable tool for finding remaining missing heritability unexplained by the current GWAS, and the developed novel statistic is able to search significant interaction between SNPs across the genome. Real data analysis showed that the results of genome-wide interaction analysis can be replicated in two independent studies.

## Author Summary

It is expected that genome-wide interaction analysis can be a possible source of finding heritability unexplained by current GWAS. However, the existing statistics for testing interaction have low power for genome-wide interaction analysis. To meet challenges raised by genome-wide interactional analysis, we develop a novel statistic for testing interaction between two loci (either linked or unlinked) and validate the null distribution and the type I error rates of the new statistic through simulations. By extensive power studies we show that the developed novel statistic has much higher power to detect interaction than the classical logistic regression. To provide evidence of gene–gene interactions as a possible source of the missing heritability unexplained by the current GWAS, we performed the genome-wide interaction analysis of psoriasis in two independent studies. The preliminary results identified 44 and 211 pairs of SNPs showing significant evidence of interactions with FDR<0.001 and 0.001<FDR<0.003, respectively, which were common in two independent studies. These included five interacting pairs of SNPs, some of which were located in the target sites: LST1/NCR3, CXCR5/BCL9L and GLS2 of miR-324-3p, miR-433, and miR-382, and 15 pairs of interacting SNPs that had nonsynonymous substitutions.

## Introduction

In the past three years, about 400 genome-wide association studies (GWAS) that focused largely on individually testing the associations of single SNP with diseases have been conducted [1]. These studies have identified more than 531 SNPs associated with different traits or diseases [2] and have provided substantial information for understanding disease mechanisms. Despite the progress that has been made, the significant SNP associations identified by GWAS account for only a few percent of the genetic variance which begs the question where and how the missing heritability can be identified [3], [4]. Possible explanations include [1], [4]:

1. The previous GWAS are mainly based on the common disease, common variant hypothesis. However, in addition to single nucleotide polymorphisms (SNPs) with a minor allele frequency (MAF) greater than 1%, there are other classes of human genetic variation including: (a) rare variants that are defined as mutations with a MAF of less than 1% and (b) structural variants including copy number variants (CNVs) and copy neutral variation such as inversions and translocations. Common diseases can also be caused by multiple rare mutations, each with a low marginal genetic effect. A more realistic model is that the entire spectrum of genetic variants ranging from rare to common contributes to disease susceptibility.
2. Most of current GWAS have focused on SNP analysis in which each variant is tested for association individually. However, common disease often arises from the combined effect of multiple loci within a gene or interaction of multiple genes within a pathway. If we only consider the most significant SNPs, the genetic variants that jointly have significant impact on risk, but individually make only a small contribution, will be missed.
3. The power of the widely used statistics for detection of gene-gene interaction and gene-environment interactions is low. Many interacting SNPs have not been identified.

Another way to discover the missing heritability of complex diseases is to investigate gene-gene and gene-environment interaction. Disease development is a dynamic process of gene-gene and gene-environment interactions within a complex biological system which is organized into interacting networks [5]. Modern complexity theory assumes that the complexity is attributed to the interactions among the components of the system, therefore, interaction has been considered as a sensible measure of complexity of the biological systems. The more interactions between the components there are, the more complex the system is. The disease may be caused by joint action of multiple loci. Motivation for studying statistical interaction is to provide increased power for detecting joint acting effects of interacting loci than testing for only marginal association of each of the loci individually. Screening for only main effects might miss the vast majority of the genetic variants that interact with each other and with environment to cause diseases [6]. We argue that the interactions hold a key for dissecting the genetic structure of complex diseases and elucidating the biological and biochemical pathway underlying the diseases [7], [8]. Ignoring gene-gene and gene-environment interactions will likely obscure the detection of genetic effects and may lead to inconsistent association results across studies [9], [10].

GWAS in which several hundred thousands or even a millions of SNPs are typed in thousands of individuals provide unprecedented opportunities for systematic exploration of the universe of variants and interactions in the entire genome and also raise several serious challenges for genome-wide interaction analysis. The first challenge comes from the problems imposed by multiple testing. Even for investigating pair-wise interaction, the total number of tests for interaction between all possible SNPs across the genome will be extremely large. Bonferroni-corrected P-values for ensuring genome-wide significance level of 0.05 will be too small to reach. The second challenge is the need for computationally simple statistics for testing interactions. The simplest way to search for interactions between two loci is to test all possible two-locus interactions. This exhaustive search demands large computations. Therefore, the computational time of each two-locus interaction test should be short. The third challenge is the power of the statistics for testing interaction. To ensure the genome-wide significance, the statistics should have high power to detect interaction. Developing simple and efficient analytic methods for evaluation of the gene-gene interactions is critical to the success of genome-wide gene-gene interaction analysis. Finally, the fourth challenge is replication of the finding of such interactions in independent studies.

This report will attempt to meet these challenges, at least in part. To achieve this, we first should define a good measure of gene-gene interaction. Despite current enthusiasm for investigation of gene-gene interactions, published results that document these interactions in humans are limited and the essential issue of how to define and detect gene-gene interactions remains unresolved. Over the last three decades, epidemiologists have debated intensely about how to define and measure interaction in epidemiologic studies [7], [8], [11]–[15]; The concept of gene-gene interactions is often used, but rarely specified with precision [16]. In general, statistical gene-gene interaction is defined as departure from additive or multiplicative joint effects of the genetic risk factors [17]. It is increasingly recognized that statistical interactions are scale dependent [18]. In other words, how to define the effects of a risk factor and how to measure departure from the independence of effects will greatly affect assessment of gene-gene interaction. The most popular scale upon which risk factors are measured in case-control studies is odds-ratio. The traditional odds-ratio is defined in terms of genotypes at two loci. Similar to two-locus association analysis where only genotype information at two loci is used, odds-ratio defined by genotypes for testing interaction will not employ allelic association information. However, it is known that interaction between two loci will generate allelic associations in some circumstances [19]. Since they do not use allelic association information between two loci, the statistical methods based on the odds-ratio that is defined in terms of genotypes will have less power to detect interaction. To overcome this limitation, we will define odds-ratio in terms of a pseudohaplotype (which is defined as two alleles located on the same paternal or maternal chromosomes) for measuring interaction, and then we will investigate its properties and develop a statistic based on pseudohaplotype defined odds-ratio for testing interaction between two loci (either linked or unlinked).

To demonstrate that the pseudohaplotype odds-ratio interaction measure-based statistic for detection of interaction between two loci will not cause false positive problems, we then investigate type I error rates. To reveal the merit and limitation of the pseudohaplotype odds-ratio interaction measure-based statistic for detection of interaction, we will compare its power for detecting interaction with the traditional logistic regression and “fast-epistasis” in PLINK [20].

Although nearly 400 GWAS have been documented, few genome-wide interaction analyses have been performed and few findings of significant interaction reported [8], [21], [22]. Emily et al [23] tested about 3,107,904–3,850,339 pairs of SNPs located in genes with potential protein-protein interaction and reported four significant cases of interactions, one in each of Crohn's Disease, bipolar disorder, hypertension and rheumatoid arthritis in the WTCCC dataset, but these have not been replicated. To further evaluate the performance of our new statistic and test the feasibility of genome-wide interaction analysis, the presented statistic was applied to interaction analysis of two independent GWAS datasets of psoriasis where 1,266,378,301 pairs of SNPs from 50,327 SNPs in the first dataset and 1,243,782,750 pairs of SNPs from 49,876 SNPs in the second dataset were tested for interactions. These SNPs in the datasets were selected from 501 pathways assembled from KEGG [24] and Biocarta (http://www.biocarta.com) pathway databases. A program for using the developed statistic to test interaction which was implemented by C++ can be downloaded from our website http://www.sph.uth.tmc.edu/hgc/faculty/xiong/index.htm.

## Methods

A case-control study design for detection of interaction between two loci (SNPs) where two loci can be either linked or unlinked were considered. The statistics for testing interaction are usually motivated by the measure of interaction. The widely used logistic regression methods for detection of gene-gene interaction are based on then odds-ratio measure of interaction. Traditional additive and multiplicative odds ratio measures of interaction are defined in terms of genotypes at two loci. In this report, a novel statistic for testing interaction between two loci is based on multiplicative odds-ratio measures defined in terms of pseudohaplotypes. For the convenience of presentation, we first briefly introduce the odds ratio interaction measure in terms of genotypes, alleles, and then present the odds ratio measure in terms of pseudohaplotypes.

### Genotype-Based Odds Ratio Multiplicative Interaction Measure

Consider two loci: G and H. Assume that the codes 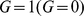 and 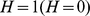 denote whether an individual is a carrier (non-carrier) of the susceptible genotypes at the loci G and H, respectively. Let D denote disease status where 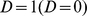 indicates an affected (unaffected) individual. Consider the following logistic model:
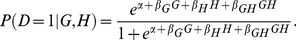
(1)The odds-ratio associated with G for nonsusceptible genotype at the locus H 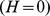 is defined as
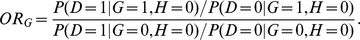
Similarly, the odds-ratio associated with H for nonsusceptible genotype at the locus G 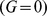 is defined as
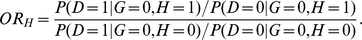
The odds-ratio associated with susceptibility at G and H compared to the baseline category 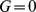 and 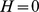 is then computed as
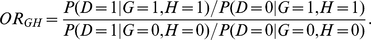
The odds for baseline category 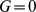 and 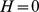 are determined as
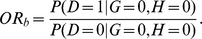
From equation (1), we clearly have
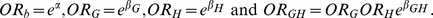
Define a multiplicative interaction measure between two loci G and H as
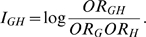
(2 – A)It is clear that
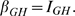
(2 – B)If 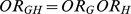, i.e., there is no interaction between loci G and H, then 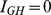. This shows that the logistic regression coefficient for interaction term 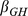 is equivalent to the interaction measure defined as log odds-ratio. The interaction measure 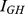 can also be written as
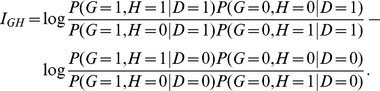



The values of odds-ratio defined in terms of genotypes depends on how to code indicator variables G and H. Suppose that alleles 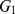 and 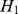 are alleles that increase disease risk. For a recessive model, G is coded as 1 if the genotype is 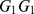, otherwise, G is coded as 0. For a dominant model, G is coded as 1 if the genotypes are either 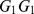 or 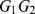, otherwise G is coded as 0. The indicator variable H can be similarly coded. However, in real data analysis, the disease models are unknown. Especially, the types of two-locus disease models are large [25]. We may have a large number of possible coding, and many of them may have larger numbers of degrees of freedom than the allelic model.

### Allele-Based Odds Ratio Multiplicative Interaction Measure

Similar to the odds ratio for genotypes, we can define odds-ratio in terms of alleles. Let 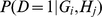 be the probability that an individual becomes affected given they have genotype 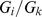 at locus G and 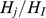 at locus H, where 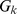 is either 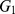 or 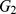 (i.e. 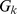 is a member of the set {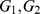}) and 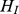 is either 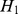 or 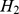 (i.e. 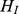 is a member of the set {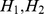}). We can similarly define 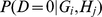. We then can determine the odds-ratio associated with the allele 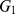 at the G locus and allele 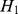 at the H locus compared to the baseline 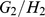 as
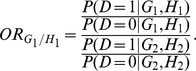
Similarly, we measure the odds-ratio associated with the alleles 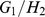 and 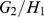, respectively as
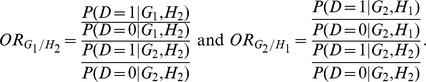
Similar to genotype, we can define a multiplicative interaction measure in terms of log odds-ratio for allele as
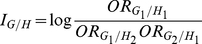
which is equivalent to
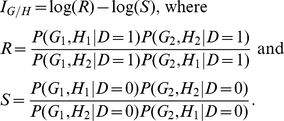
The “fast-epistasis” test statistic in PLINK (http://pngu.mgh.harvard.edu/~purcell/plink/index.shtml) is defined as
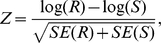
where SE(R) and SE(S) denote the standard deviation of R and S, respectively. Absence of interaction is implied if and only if
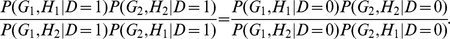
This is the basis of the “fast-epistasis” test in PLINK.

### Haplotype-Based Odds Ratio Multiplicative Interaction Measure

Suppose that the locus G has two alleles 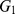 and 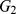 and the locus H has two alleles 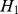 and 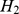. Let 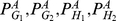 and 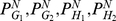 be the frequencies of the alleles 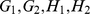 in the cases and controls, respectively. For the discussion of convenience, we introduce a terminology of “pseudohaplotype”. When two loci are linked, a pseudohaplotype is defined as the regular haplotype. When two loci are unlinked, a pseudohaplotype is defined as a set of alleles that are located in the same paternal or maternal chromosomes. The frequencies of a pseudohaplotype can be estimated by the classical methods for estimation of haplotype frequencies such as Expectation Maximization (EM) Algorithms. For simplicity, hereafter we will not make distinction between the haplotype and pseudohaplotype. When two loci are unlinked, a haplotype is understood as a pseudohaplotype. Let 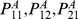, 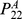 and 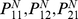, 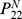 denote the frequencies of haplotypes 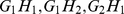 and 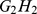 in the cases and controls, respectively. We define a penetrance of the haplotype 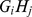 as the probability that an individual becomes affected given they have phased genotype 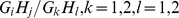. Let 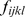 be the penetrance of an individual with the genotype 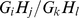, 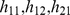 and 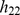 be the penetrance of the haplotypes 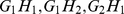 and 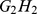, respectively. The penetrance of the haplotype 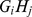 can be mathematically defined as
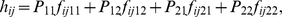
where 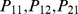 and 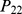 are the population frequencies of the haplotypes 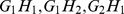 and 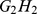, respectively.


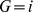
 and 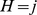 represent a genotype coding scheme. Their represented genotypes depend on the specific genotype coding scheme. It should be noted that the haplotype 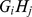 and 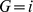 and 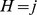 have different meanings. By the same idea in defining genotype-based odds ratio in terms of penetrance of combinations of genotypes, we can determine the odds-ratio associated with the haplotypes 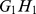 compared to the baseline haplotype 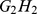 in terms of penetrance of the haplotypes as
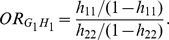
Similarly, we calculate the odds-ratio associated with the haplotypes 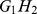 and 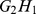, respectively, as
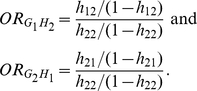
It is noted that replacing 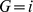 and 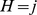 in the definition of odds-ratio in terms of genotypes by 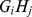 leads to the definition of odds-ratio based on the haplotypes. However, the values and biological meanings of these two types of odds-ratios are different.

Similar to genotypes, we can compute a multiplicative interaction measure in terms of log odds-ratio for haplotypes as
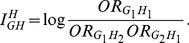
(3)In the absence of interaction, we have
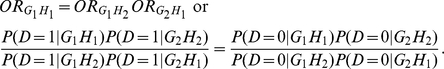
The multiplicative odds-ratio interaction measure in equation (3) is defined by the penetrance of the haplotypes. From case-control data it is difficult to calculate the penetrance of the haplotypes. However, we can show that the multiplicative odds-ratio interaction measure in equation (3) can be reduced to (Text S1, Appendix A)
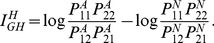
(4)There are many algorithms and software to infer the haplotype frequencies in cases and controls. Therefore, we can easily calculate the multiplicative odds-ratio interaction measure by equation (4). It can be seen from equation (4) that the absence of interaction between two loci occurs if and only if the ratio of haplotypes frequencies 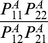 in the cases and the ratio of haplotypes frequencies 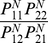 in the controls are equal.

To gain understanding the multiplicative odds-ratio interaction measure, we study several special cases.

#### Case 1

One of two loci is a marker. If we assume that the locus H is a marker and is not associated with disease, then we have
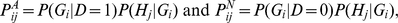
which implies that
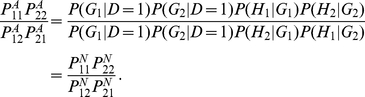
Thus, we obtain 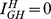. In other words, if the locus H is a marker, there is no interaction between two loci G and H. The interaction measure 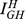 between two loci should be equal to zero. Hence, our multiplicative odds-ratio interaction measure correctly characterizes the marker case.

#### Case 2

Logistic regression interpretation.

We define two indicator variables:
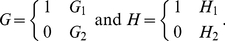
(5)Then four haplotypes at two loci can be coded as follows:[Table pgen-1001131-t009]

**Table pgen-1001131-t009:** 

	G	H
G_1_H_1_	1	1
G_1_H_2_	1	0
G_2_H_1_	0	1
G_2_H_2_	0	0

It follows from the logistic regression model in equation (1) that
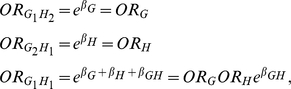
where odds-ratios 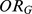 and 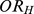 are defined in terms of alleles, i.e.
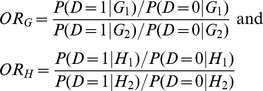
Therefore, the haplotype multiplicative odds-ratio interaction measure 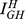 is equal to 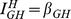, which has the same form as that in equation (2-B). This indicates that if the coding for the genotypes in the genotype multiplicative odds-ratio interaction measure 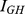 is replaced by the coding for the haplotypes in equation (5) then we can obtain the haplotype multiplicative odds-ratio interaction measure.

### Test Statistics

In the previous section we defined the haplotype multiplicative odds-ratio interaction measure, which can be estimated by haplotype frequencies in cases and controls. By the delta method, we can obtain the variance of the estimator of the haplotype odds-ratio interaction measure [26]:
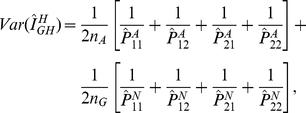
where 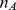 and 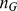 are the number of sampled individuals in cases and controls. By the standard asymptotic theory we can define the haplotype odds-ratio interaction measure-based statistic for testing interaction between two loci:
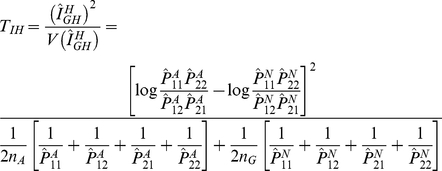
(6)where 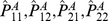 and 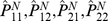 are the estimators of the corresponding haplotype frequencies in cases and controls, respectively. When sample sizes are large enough to ensure application of large sample theory, 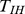 is asymptotically distributed as a central 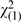 distribution under the null hypothesis of no interaction between two loci. Under an alternative hypothesis of of interaction between two loci being present, the statistic 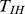 is asymptotically distributed as a noncentral 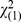 distribution with noncentrality parameter proportional to the haplotype multiplicative odds-ratio interaction measure. This statistic can be applied to both linked and unlinked loci. As we explained in Text S1, Appendix B, the proposed statistic 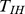 is different from the “fast-epistasis” test in PLINK.

For the unlinked loci, we can use case only design [27], [28] to study interaction between two loci in which equation is reduced to
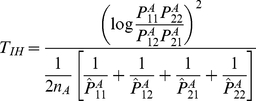
(7)


## Results

### Null Distribution of Test Statistics

**Figure 1:**
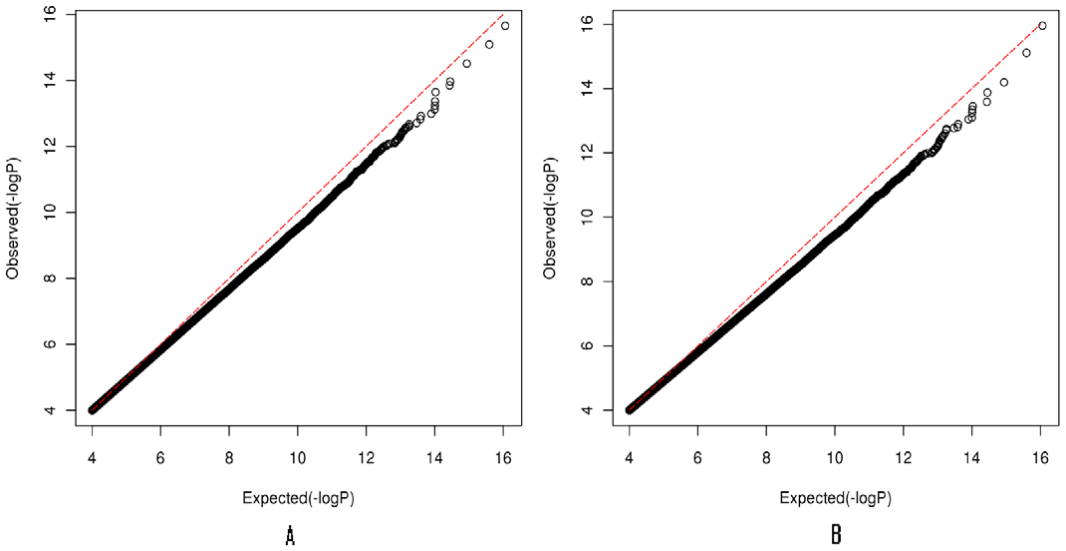
Quantile-quantile plots for the test statistic 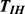. (A) Quantile-quantile plots for the test statistic 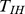 in dataset 1. The P-values (<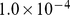) for the test are plotted (as −log10 values) as a function of its expected p values. (B) Quantile-quantile plots for the test statistic 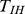 in dataset 2. The P-values (<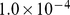) for the test are plotted (as −log10 values) as a function of its expected p values.

In the previous sections, we have shown that when the sample size is large enough to apply large sample theory, the distribution of the statistic 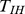 for testing the interaction between two loci under the null hypothesis of no interaction between them is asymptotically a central 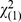 distribution. To examine the validity of this statement, we performed a series of simulation studies. MATLAB was used to generate two-locus genotype data of the sample individuals. A total of 100,000 individuals from a general population with an allele frequency 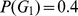, 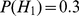, haplotype frequency 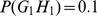 and disequilibrium coefficient 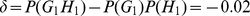 were generated. A total of 10,000 simulations were repeated. Type I error rates were calculated by random sampling 500–1,000 individuals as cases and controls from the general population. Table 1 and Table 2 show that the estimated type I error rates of the statistic 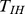 for testing interaction between two loci, assuming 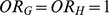 and 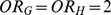, were not appreciably different from the nominal levels 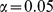, 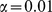 and 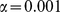. To further examine the validity of the test statistic, we constructed Quantile-quantile (Q-Q) plots of the test statistic in datasets 1 and 2 shown in Figures 1A and 1B, where the P-values of the tests were plotted (as −log10 values) as a function of p values from the expected null distribution. Since the total number of all possible pair-wise tests for interaction between SNPs is too large to store all the results in computer we only stored P-values <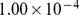. Consequently, Q-Q plots started with 4. Figures 1A and 1B showed good agreement with the null distribution.

**Table 1:** Type I error rates of the statistic 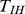 to test for interaction between two loci, assuming 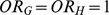.

Sample Size	Nominal levels		
	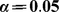	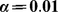	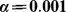
300	0.04790	0.00995	0.00080
400	0.04815	0.00820	0.00080
500	0.04745	0.00930	0.00085
600	0.04880	0.00850	0.00095
700	0.05060	0.00920	0.00075
800	0.05120	0.01015	0.00100
900	0.04935	0.00805	0.00090
1000	0.04860	0.00880	0.00090

**Table 2:** Type I error rates of the statistic 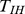 to test for interaction between two loci, assuming 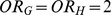.

Sample Size	Nominal levels		
	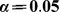	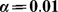	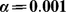
300	0.04990	0.00945	0.00120
400	0.04995	0.01030	0.00085
500	0.05170	0.01065	0.00080
600	0.05070	0.00980	0.00100
700	0.04725	0.00965	0.00113
800	0.04945	0.00895	0.00075
900	0.04830	0.00950	0.00080
1000	0.04920	0.00975	0.00110

**Table 3:** Two-locus disease models.

Recessive 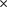 Recessive			
Locus 1\2	D_2_D_2_	D_2_d_2_	d_2_d_2_
D_1_D_1_	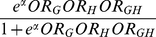	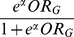	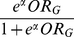
D_1_d_1_	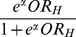	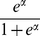	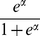
d_1_d_1_	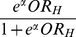	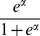	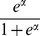

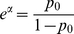
, 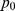 is the prevalence of the disease in the population.

The elements in the Table are the penetrance as a function of the joint genotype at loci 1 and 2 with rows indexing genotype at locus 1 and columns indexing genotype at locus 2.

### Power Evaluation

**Figure 2:**
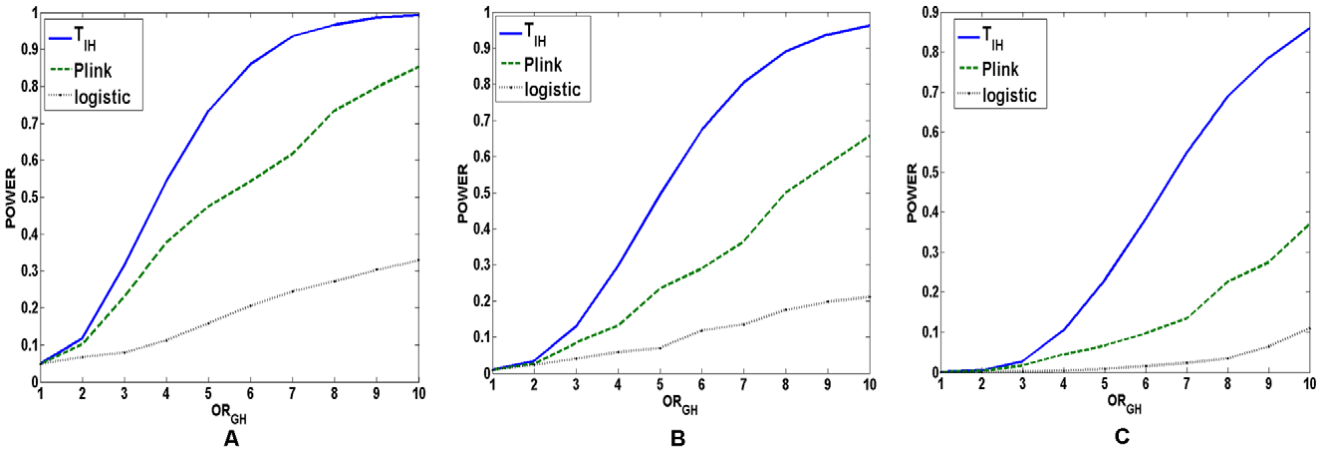
Power of the statistics for testing interaction between two linked loci under recessive disease model. (A) The power of the test statistic 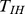, the “fast-epistasis” in PLINK and logistic regression analysis for testing interaction between two linked loci as a function of traditional odds-ratio 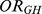 under a two-locus recessive
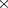
recessive disease model, where the number of individuals in both the case and control groups is 2,000, the significance level is 0.05, and the odds-ratios at two loci were 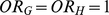. (B) The power of the test statistic 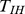, the “fast-epistasis” in PLINK and logistic regression analysis for testing interaction between two linked loci as a function of traditional odds-ratio 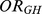 under a two-locus recessive
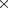
recessive disease model, where the number of individuals in both the case and control groups is 2,000, the significance level is 0.01, and the odds-ratios at two loci were 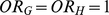. (C) The power of the test statistic 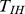, the “fast-epistasis” in PLINK and logistic regression analysis for testing interaction between two linked loci as a function of traditional odds-ratio 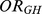 under a two-locus recessive
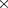
recessive disease model, where the number of individuals in both the case and control groups is 2,000, the significance level is 0.001, and the odds-ratios at two loci were 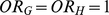.

**Figure 3:**
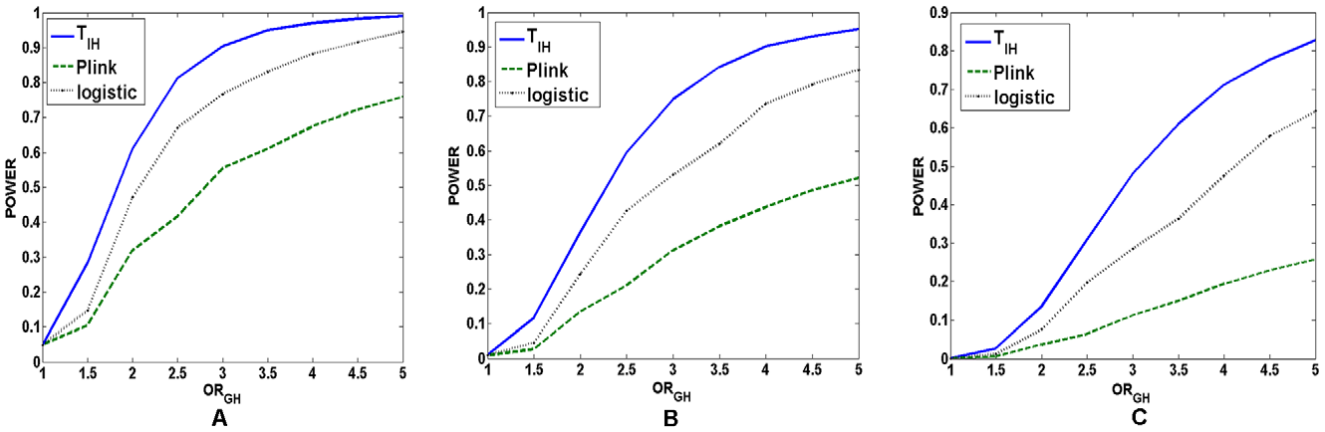
Power of the statistics for testing interaction between two linked loci under dominant disease model. (A) The power of the test statistic 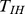, the “fast-epistasis” in PLINK and logistic regression analysis for testing interaction between two linked loci as a function of traditional odds-ratio 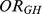 under a two-locus dominant
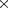
dominant disease model, where the number of individuals in both the case and control groups is 1,000, the significance level is 0.05, and the odds-ratios at two loci were 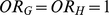. (B) The power of the test statistic 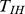, the “fast-epistasis” in PLINK and logistic regression analysis for testing interaction between two linked loci as a function of traditional odds-ratio 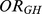 under a two-locus dominant
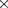
dominant disease model, where the number of individuals in both the case and control groups is 1,000, the significance level is 0.01, and the odds-ratios at two loci were 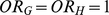. (C) The power of the test statistic 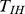, the “fast-epistasis” in PLINK and logistic regression analysis for testing interaction between two linked loci as a function of traditional odds-ratio 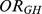 under a two-locus dominant
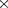
dominant disease model, where the number of individuals in both the case and control groups is 1,000, the significance level is 0.001, and the odds-ratios at two loci were 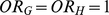.

**Figure 4:**
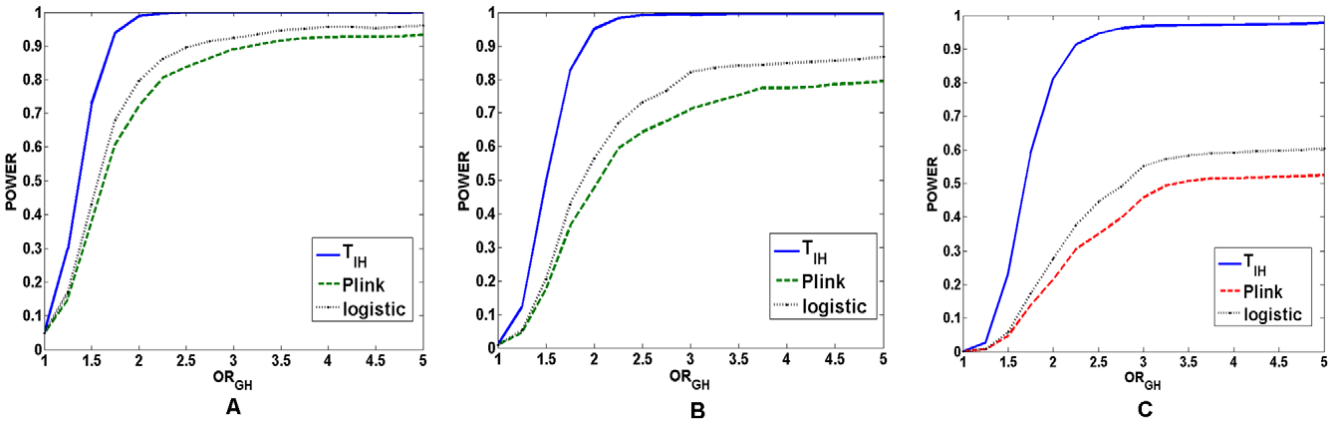
Power of the statistics for testing interaction between two linked loci under additive disease model. (A) The power of the test statistic 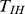, the “fast-epistasis” in PLINK and logistic regression for testing interaction between two linked loci analysis as a function of traditional odds-ratio 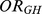 under a two-locus additive
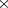
additive disease model, where the number of individuals in both the case and control groups is 1,000, the significance level is 0.05, and the odds-ratios at two loci were 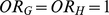. (B) The power of the test statistic 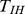, the “fast-epistasis” in PLINK and logistic regression for testing interaction between two linked loci analysis as a function of traditional odds-ratio 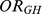 under a two-locus additive
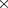
additive disease model, where the number of individuals in both the case and control groups is 1,000, the significance level is 0.01, and the odds-ratios at two loci were 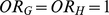. (C) The power of the test statistic 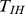, the “fast-epistasis” in PLINK and logistic regression for testing interaction between two linked loci analysis as a function of traditional odds-ratio 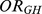 under a two-locus additive
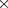
additive disease model, where the number of individuals in both the case and control groups is 1,000, the significance level is 0.001, and the odds-ratios at two loci were 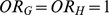.

To evaluate the performance of the statistic 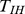 for detection of interaction between two loci, we compared the power of the statistic 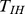 to that of the logistic model and the “fast epistasis” test in PLINK. Power was calculated by simulation. A total of 1,000,000 individuals from a general population with allele frequencies 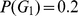, 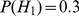 and 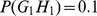 and disequilibrium coefficient 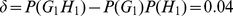 were generated. Two-locus disease models were used to generate cases and controls, and summarized in Table 3 where odds-ratio was defined in terms of genotypes. We considered three types of genotype coding. For a recessive model, homozygous wild type, heterozygous, and homozygous risk increasing genotypes were coded as 0, 0, 1, respectively. For a dominant model, homozygous wild type, heterozygous, and homozygous risk increasing genotypes were coded as 0, 1, and 1, respectively. For an additive model, they were coded as 0, 1, and 2, respectively. The genotype coding for the logistic regression matched the simulation model. The statistic 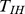 in equation (6) for the case-control version was used to evaluate the power. In the power simulations, we also assumed that 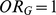 and 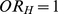. An individual who is randomly sampled from the general population was assigned to case or control status depending on the two-locus disease models in Table 3. The process was repeated until a sample of 1,000 cases and 1,000 controls for the dominant and additive models, or a sample of 2,000 cases and 2,000 controls for the recessive model was obtained. A total of 10,000 simulations were repeated. In Figures 2A–2C, power comparisons among the logistic regression model, the “fast-epistasis” in PLINK and the statistic 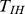 under two-locus recessive
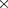
recessive disease model for significance levels 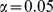, 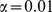 and 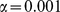, respectively are presented. In Figures 3A–3C, power comparisons among the logistic regression model, the “fast-epistasis” in PLINK and the statistic 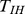 under two-locus dominant
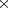
dominant disease model for significance levels 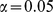, 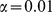 and 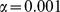, respectively are shown. In Figures 4A–4C, power comparisons between the logistic regression model and the statistic 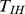 under two-locus additive
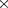
additive disease model for significance levels 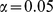, 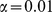 and 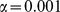, respectively are demonstrated. Several remarkable features emerge from these Figures. First, these power Figures indeed demonstrate that the power increases as the measure of the interaction between two loci increases. The power curves were plotted as a function of the traditional genotype odds ratio 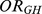. We observed that the power curves were a monotonic increasing function of the genotype odds ratio 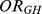. Therefore, the test statistic 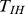 can detect the strength of the interaction between two loci. Second, the test statistic 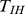 had much higher power to detect interaction between two loci than the logistic regression and the “fast-epistasis” test in PLINK. Third, the more complex the disease models were, the larger the differences in power between the test statistic 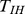, the “fast-epistasis” test in PLINK and logistic regression that were observed.

**Table 4:** Interacting SNPs with non-synonymous mutation.

SNP1(rs)	Gene1	SNP2(rs)	Gene2	Dataset 1		Dataset 2		Nonsynonymous mutation	Protein Residue
				P-Value	FDR	P-Value	FDR		
10837771	OR51B4	16973321	RYR3	1.20E-07	9.00E-04	2.28E-07	1.34E-03	rs10837771	T
7671095	GRID2	10839659	OR2D3	2.00E-08	3.97E-04	2.82E-08	5.29E-04	rs10839659	S
1545133	POLR1B	8064077	MYH11	6.71E-07	1.97E-03	5.88E-07	2.06E-03	rs1545133	L
1958715	OR4L1	3844750	EFNA5	2.15E-08	4.10E-04	1.25E-07	1.03E-03	rs1958715	N
1958716	OR4L1	3844750	EFNA5	4.48E-08	5.73E-04	1.22E-07	1.02E-03	rs1958716	V
2227956	HSPA1L	3135392	HLA-DRA	3.20E-10	6.02E-05	7.82E-10	1.05E-04	rs2227956	M
2227956	HSPA1L	3134929	NOTCH4	7.76E-09	2.57E-04	2.36E-11	2.07E-05	rs2227956	M
1799964	LTA/TNF	2227956	HSPA1L	7.52E-09	2.53E-04	2.98E-08	5.42E-04	rs2227956	M
1052248	LST1/NCR3	2227956	HSPA1L	8.24E-07	2.17E-03	1.87E-08	4.40E-04	rs2227956	M
35258	PDE4D	2230793	IKBKAP	7.50E-08	7.24E-04	7.85E-07	2.34E-03	rs2230793	L
2254524	LSS	10860869	IGF1	8.58E-08	7.70E-04	6.35E-09	2.71E-04	rs2254524	V
327325	NRG1	3742290	UTP14C	7.47E-07	2.07E-03	5.90E-07	2.06E-03	rs3742290	A
4253211	ERCC6	10435892	GABBR2	5.60E-07	1.81E-03	1.11E-09	1.23E-04	rs4253211	P
940389	STON1	10745676	PLXNC1	2.20E-08	4.14E-04	7.02E-07	2.23E-03	rs940389	T
676925	CXCR5	999890	PIP5K3	3.07E-07	1.38E-03	2.93E-07	1.51E-03	rs999890	A

**Figure 5:**
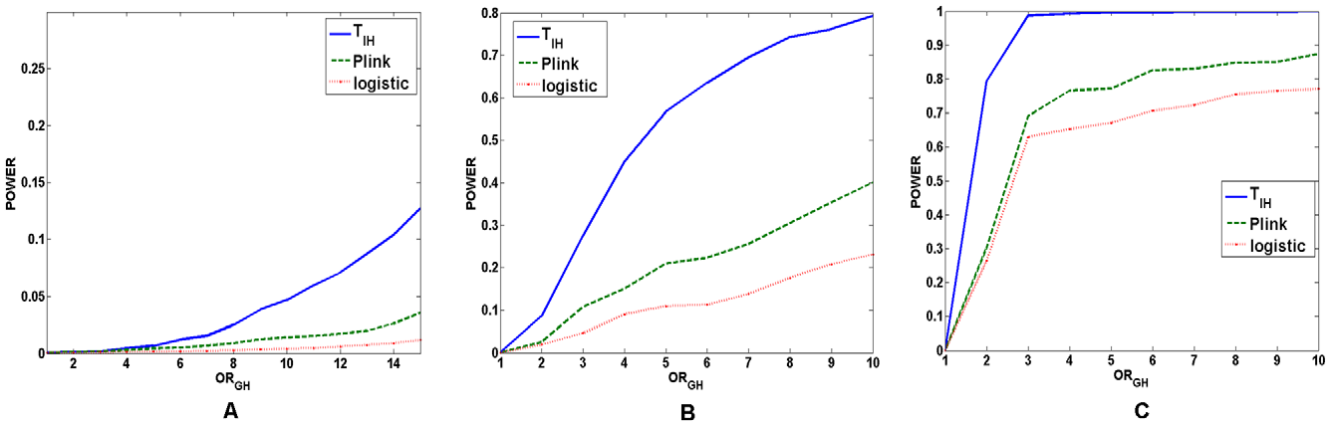
Power of the statistics for testing interaction between two unlinked loci. (A) The power of the test statistic 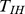, the “fast-epistasis” in PLINK and logistic regression analysis for testing interaction between two unlinked loci as a function of traditional odds-ratio 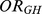 under a two-locus recessive
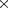
recessive disease model, where the number of individuals in both the case and control groups is 2,000, the significance level is 0.001, and the odds-ratios at two loci were 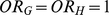. (B) The power of the test statistic 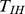, the “fast-epistasis” in PLINK and logistic regression analysis for testing interaction between two unlinked loci as a function of traditional odds-ratio 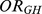 under a two-locus dominant
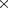
dominant disease model, where the number of individuals in both the case and control groups is 1,000, the significance level is 0.001, and the odds-ratios at two loci were 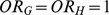. (C) The power of the test statistic 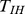, the “fast-epistasis” in PLINK and logistic regression analysis for testing interaction between two unlinked loci as a function of traditional odds-ratio 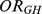 under a two-locus additive
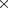
additive disease model, where the number of individuals in both the case and control groups is 1,000, the significance level is 0.001, and the odds-ratios at two loci were 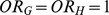.

When two loci are unlinked where we do not observe the allelic association between two loci in the population as a whole, our results also hold. We assumed the following allele and haplotype frequencies in the population: 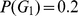, 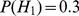 and 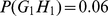. Other parameters were defined as before. A total of 10,000 simulations were repeated to simulate the power of three statistics under three disease models with the significance level 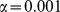. Figures 5A, 5B and 5C showed the power of three statistics for testing interaction between two unlinked loci under two-locus recessive
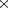
recessive, dominant
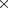
dominant, and additive
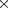
additive disease models, respectively. These Figures again demonstrated that the power of the test statistic 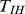 was still much higher than that of the logistic regression and the “fast-epistasis” test in PLINK. The conclusions still hold for the significance levels 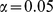 and 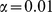 (Data were not shown).

### Application to Pathway-Based Genome-Wide Interaction Analysis of Psoriasis

To evaluate its performance for detection of interaction between two loci, the proposed test statistic 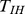 was applied to interaction analysis of two independent GWAS datasets of psoriasis which were downloaded from dbGaP. Psoriasis is a common chronic inflammatory skin disease affecting 2%–3% of the world population. Originally, the first study included 955 individuals with psoriasis and 693 controls, which is considered as dataset 1. The second replication study included 466 individuals with psoriasis and 732 controls, which is designated dataset 2. All cases and controls are of European origin [29]–[31]. After using PLINK [20] to check for contamination, cryptic family relationship and non-Caucasian ancestry, 123 samples were excluded. Subsequently we retained for analysis 915 cases and 675 controls from the first study and 431 cases and 702 controls from the second study. All 2,723 samples had been genotyped with the Perlegen 500K array. In the initial dataset, 451,724 SNPs passed quality control (call rate>95%). To further ensure the quality of the typed SNPs, we used PLINK software to remove the SNPs with >5% missing genotypes, Hardy-Weinberg disequilibrium (P-values <0.0001), MAF<0.01 and duplicated markers. In this application, we only considered common SNPs with MAF>0.01. After quality control filtering, a total of 451,724 SNPs were pruned to 443,018 and 439,201 SNPs with the average genotyping rate 99.3% in the first and second studies, respectively.

Since testing for all possible two-locus interactions across the genome in genome-wide interaction analysis requires extremely large computation, we conducted pathway-based genome-wide interaction analysis. We assembled 501 pathways from KEGG [24] and Biocarta (http://www.biocarta.com). The assignment of SNPs to a gene was obtained from NCBI human9606 database (version b129). We used the statistic 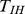 to test interactions of all possible pairs of SNPs located in genes within the assembled 501 pathways. The total number of SNPs in dataset 1 and dataset 2 being tested was 50,327 and 49,876, respectively. The serious problem in genome-wide interaction analysis is multiple testing. We used two strategies to tackle this problem. One is to use false discovery rate (FDR) [32] to declare significance of interaction. Another is replication of the findings in two independent studies, which enhances confidence in interaction tests [22]. We looked for consistent results across the two independent studies.

**Figure 6:**
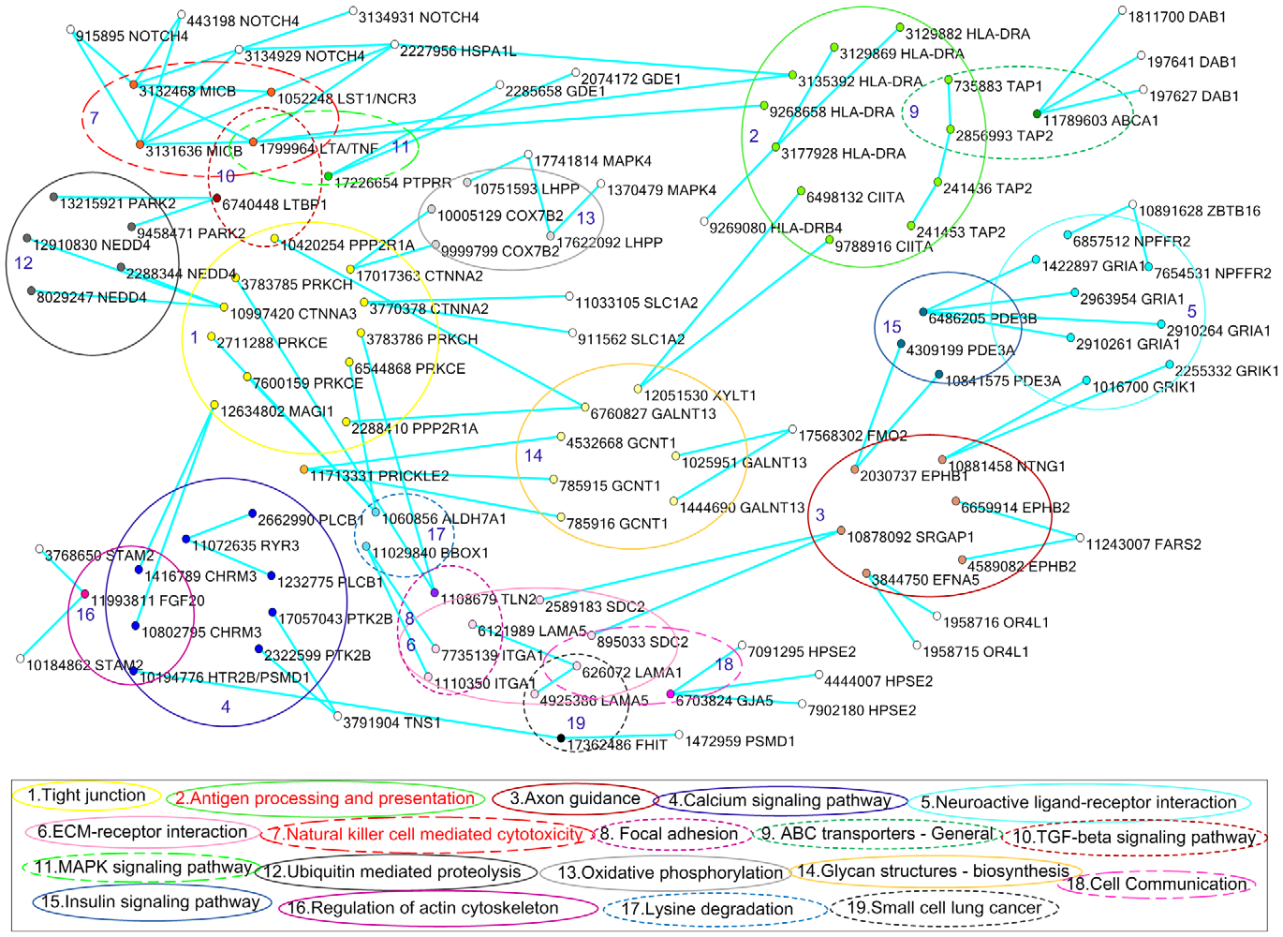
Interacting SNPs that were located in 19 pathways formed a network. Each pathway was represented by an ellipse with the number. The SNPs were represented by nodes and placed insight their located pathways. Nearby each SNP there was its RS number and the name of its located gene. The pathway and its harbored SNPs were labeled by the same color. The interacting SNPs were connected by the solid light green lines.

In total, 44 pairs of SNPs showed significant evidence of interactions with FDR<0.001, which roughly corresponds to the P-value <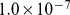, in two independent studies (Table S1). These 44 pairs of SNPs were derived from 71 distinct SNPs located in 60 genes, including HLA-C, HLA-DRA, HLA-DPA1, LST1, MICB and NOTCH4. Of 44 pairs of SNPs, only one pair of interacting SNPs: rs2395471 and rs2853950 showed significant marginal association in two independent studies. An additional 211 pairs of SNPs with FDR less than 0.003 in the two studies is listed in Table S2. These interacting SNPs were mainly located in 19 pathways, including a number of signaling pathways, and immune-related antigen processing and presentation as well as natural killer cell mediated cytotoxicity pathways (Figure 6). Several remarkable features emerge from these results. First, although we can observe a few interactions between SNPs within a gene, the majority of interactions occurred between genes that are often in different pathways. Since the number of SNPs typed within each gene was limited, it is unknown whether this is a general rule or just a special case. Second, a SNP in one gene might interact with multiple SNPs in multiple genes. For example, SNP rs3131636 in the gene MICB interacting with the SNPs rs915895, rs443198, rs3134929 in the gene NOTCH4, the SNP rs1052248 in the gene LAST1/Natural cytotoxicity triggering receptor 3 (NCR3) and the SNP rs1799964 in the gene LTA/TNF. SNP rs1799964 in the gene LTA/TNF interacting with SNPs rs3131636, rs3132468 in the gene MICB, SNPs rs9268658 and rs3135392 in the gene HLA-DRA, SNP rs2227956 in the gene HSPA1L. However, this does not imply that multiple causal SNPs within a gene will interact with multiple causal SNPs within another gene. It is quite likely that this is due to LD between the SNPs within a gene. Third, although interacting SNPs did not form large connected networks, the interacting SNPs connected pathways into a large complicated network. This may imply that many genes and pathways are involved in the development of psoriasis. Fourth, upstream of many pathways included genes with interacting SNPs. For example, genes MICB, CHRM3, HLA-DRA and CIITA, EPHB1 and EPHB2, LAMA1 and LANA5, ITGA1, LTBP1, TNF, and FGF20 that contain interacting SNPs are in the upstream of natural killer cell mediated cytotoxicity, calcium signaling pathway, antigen processing and presentation, axon guidance, ECM-receptor interaction pathway, focal adhesion, TGFB pathway, MAPK pathway and regulation of acting cytoskeleton, respectively. Fifth, most interacting SNPs are in introns and accounted for 77% of total interacting SNPs.

Table 4 listed 15 pairs of interacting SNPs that have non-synonymous substitutions. It is unknown how these nonsynonymous mutations are involved in the pathogenesis of psoriasis. From the literature we know that Plexin C1 receptor is a tumor suppressor gene for melanoma [33], NOTCH4 is involved in schizophrenia [34], Phosphodiesterase 4D (PDE4D) is associated with ischemic stroke [35], HLA-DRA is one of the HLA class II alpha chain genes that plays a central role in antigen processing, and neuregulin 1 (NRG1) has been implicated in diseases such as cancer, schizophrenia and bipolar disorder [36].

**Table 5:** Five pairs of interacting SNPs, one of which falls in the microRNA binding region.

SNP1(rs)	Gene 1	SNP2(rs)	Gene 2	Dataset 1		Dataset 2		MicroRNA Binding Site
				P-Value	FDR	P-Value	FDR	
1052248	LST1/NCR3	2227956	HSPA1L	8.24E-07	2.17E-03	1.87E-08	4.40E-04	rs1052248 (miR-324-3p)
1052248	LST1/NCR3	3131636	MICB	5.56E-13	3.03E-06	7.76E-10	1.04E-04	rs1052248 (miR-324-3p)
676925	CXCR5/BCL9L	999890	PIP5K3	3.07E-07	1.38E-03	2.93E-07	1.51E-03	rs676925 (miR-382)
163274	ACSM1	2638315	GLS2	8.16E-07	2.16E-03	9.14E-07	2.51E-03	rs2638315 (miR-433)
2072619	MYH11	3822711	GALNT10	1.83E-07	1.09E-03	3.77E-08	6.03E-04	rs3822711 (miR-324-3p)

Table 5 includes five interacting pairs of SNPs, one of which falls in the microRNA (MiRNA) binding region. miRNAs, which are 22 nucleotide small RNAs and regulate gene expressions by pairing the miRNA seed region with the target sites, have been implicated in many biological processes including the immune response, biogenesis and tumorigenesis [37]. Mutations in the target sites will affect miRNA activity. A number of studies have identified polymorphisms in the miRNA target sites associated with the diseases [37]. Interestingly, we identified four SNPs in the miRNA (miR-324-3p, miR-433, and miR-382) target sites which interact with five SNPs to contribute to psoriasis. In previous studies, miR-382 has been associated with dermatomyositis, Duchenne muscular dystrophy and Miyoshi myopathy [38], miR-433 and miR-324 with lupus nephritis [39] and miR-433 with Parkinson's disease [40].

**Table 6:** Top 20 pairs of interacting SNPs.

	Association of SNP							Interaction			
	P-value				P-value			Dataset 1		Dataset 2	
SNP1(rs)	Dataset 1	Dataset 2	Gene 1	SNP2(rs)	Dataset 1	Dataset 2	Gene 2	P-Value	FDR	P-Value	FDR
626072	0.227074	0.053394	LAMA1	6121989	0.862496	0.311346	LAMA5	1.11E-15	1.41E-07	5.67E-07	2.03E-03
626072	0.227074	0.053394	LAMA1	4925386	0.935809	0.264641	LAMA5	1.47E-13	1.73E-06	9.81E-07	2.59E-03
1052248	1.28E-05	0.002907	LST1/NCR3	3131636	0.012961	0.0006472	MICB	5.56E-13	3.03E-06	7.76E-10	1.04E-04
1052248	1.28E-05	0.002907	LST1/NCR3	3132468	0.014008	0.0005969	MICB	8.41E-13	3.70E-06	8.96E-10	1.12E-04
443198	0.000703	2.35E-11	NOTCH4	3131636	0.012961	0.0006472	MICB	1.13E-11	1.24E-05	3.98E-08	6.18E-04
443198	0.000703	2.35E-11	NOTCH4	3132468	0.014008	0.0005969	MICB	1.19E-10	3.76E-05	6.55E-08	7.72E-04
1799964	0.001104	0.009606	LTA/TNF	3131636	0.012961	0.0006472	MICB	1.62E-10	4.35E-05	1.36E-09	1.34E-04
1799964	0.001104	0.009606	LTA/TNF	3132468	0.014008	0.0005969	MICB	2.94E-10	5.80E-05	2.51E-09	1.78E-04
4766587	0.813376	0.391864	ACACB	4807055	0.530091	0.0742653	NDUFA11	3.07E-10	5.90E-05	6.61E-07	2.17E-03
2227956	0.001216	0.000149	HSPA1L	3135392	0.581239	0.75373	HLA-DRA	3.20E-10	6.02E-05	7.82E-10	1.05E-04
1060856	0.824965	0.751351	ALDH7A1	2711288	0.258241	0.0910624	PRKCE	3.57E-10	6.33E-05	2.56E-07	1.42E-03
326346	0.979881	0.212752	CD47	11081513	0.229512	0.79174	VAPA	4.24E-10	6.84E-05	2.81E-07	1.48E-03
1932067	0.043627	0.970441	PAFAH2	13203100	0.208767	0.598145	TIAM2	5.64E-10	7.81E-05	3.04E-08	5.47E-04
2012359	0.369854	0.40799	PARP4	10823333	0.614239	0.882698	HK1	5.65E-10	7.82E-05	7.20E-07	2.25E-03
9311951	0.131357	0.719726	MAGI1	11195879	0.361463	0.072601	NRG3	8.53E-10	9.50E-05	8.25E-07	2.40E-03
3768650	0.318227	0.611621	STAM2	11993811	0.732675	0.862927	FGF20	9.20E-10	9.81E-05	3.25E-08	5.64E-04
785915	0.290127	0.406203	GCNT1	11713331	0.752161	0.621571	PRICKLE2	1.30E-09	1.15E-04	2.95E-07	1.51E-03
785916	0.274961	0.307539	GCNT1	11713331	0.752161	0.621571	PRICKLE2	1.37E-09	1.17E-04	5.51E-07	2.00E-03
1202674	0.254783	0.978976	RPS6KA2	6061796	0.952187	0.932697	CDH4	2.46E-09	1.52E-04	1.63E-08	4.14E-04
1048471	0.414631	0.566854	ST3GAL1	2830096	0.754145	0.728396	APP	2.95E-09	1.65E-04	9.63E-07	2.57E-03

Some researchers suggest that in genome-wide interaction analysis only SNPs with large or mild marginal genetic effects should be tested for interaction. To examine whether this strategy will miss detection of interacting SNPs, we showed in Table 6 the 20 top pairs of interacting SNPs and in Table S3 all pairs of interacting SNPs with FDR less than 0.003. Surprisingly, 75% of SNPs with P-values (in dataset 1) larger than 0.2 and 44% of SNPs with P-values larger than 0.5 in two studies were observed in Table S3. Table 6 and Table S3 strongly demonstrated that while both SNPs did not demonstrate significant evidence of marginal association, they did show significant evidence of interaction.

**Table 7:** P-values of 20 pairs of interacting SNPs calculated by the statistic TIH, PLINK, and logistic regression coded by genotypes.

				P-Value									
				Dataset1					Dataset2				
				T_IH_	PLINK	Logistic Regression			T_IH_	PLINK	Logistic Regression		
rs1	Gene 1	rs2	Gene 2			Recessive	Additive	Dominant			Recessive	Additive	Dominant
626072	LAMA1	6121989	LAMA5	1.11E-15	1.63E-07	2.61E-02	7.82E-08	3.14E-06	5.67E-07	0.001854	3.65E-02	1.40E-03	1.10E-01
626072	LAMA1	4925386	LAMA5	1.47E-13	1.01E-06	7.64E-02	5.82E-07	1.05E-05	9.81E-07	0.002709	5.07E-02	1.88E-03	1.27E-01
1052248	LST1/NCR3	3131636	MICB	5.56E-13	2.86E-09	8.61E-03	1.14E-09	1.11E-05	7.76E-10	1.76E-05	4.47E-01	9.67E-06	9.75E-06
1052248	LST1/NCR3	3132468	MICB	8.41E-13	4.28E-09	1.16E-02	1.72E-09	1.10E-05	8.96E-10	2.03E-05	4.75E-01	9.97E-06	7.73E-06
443198	NOTCH4	3131636	MICB	1.13E-11	6.14E-08	3.29E-01	2.95E-08	4.53E-05	3.98E-08	6.33E-05	2.93E-02	3.63E-05	7.83E-04
443198	NOTCH4	3132468	MICB	1.19E-10	2.81E-07	3.21E-01	1.52E-07	1.57E-04	6.55E-08	6.32E-05	2.81E-02	4.65E-05	1.39E-03
1799964	LTA/TNF	3131636	MICB	1.62E-10	3.08E-07	7.09E-02	1.52E-07	3.68E-02	1.36E-09	7.26E-05	4.19E-01	3.25E-05	2.59E-06
1799964	LTA/TNF	3132468	MICB	2.94E-10	7.26E-07	9.57E-02	3.80E-07	3.80E-02	2.51E-09	8.30E-05	4.51E-01	4.33E-05	2.76E-06
4766587	ACACB	4807055	NDUFA11	3.07E-10	6.25E-05	3.13E-04	4.84E-05	4.51E-01	6.61E-07	0.000855	3.27E-03	7.74E-04	9.85E-01
2227956	HSPA1L	3135392	HLA-DRA	3.20E-10	2.46E-06	1.14E-04	1.49E-06	2.63E-02	7.82E-10	9.60E-06	7.29E-05	5.17E-06	2.14E-01
1060856	ALDH7A1	2711288	PRKCE	3.57E-10	3.01E-05	1.93E-07	1.71E-05	8.81E-01	2.56E-07	0.000292	7.50E-01	2.09E-04	2.26E-04
2012359	PARP4	10823333	HK1	5.65E-10	3.84E-05	1.26E-04	2.70E-05	1.37E-01	7.20E-07	0.000148	2.06E-04	7.68E-05	1.00E+00
9311951	MAGI1	11195879	NRG3	8.53E-10	6.22E-05	1.00E-03	3.29E-05	3.40E-03	8.25E-07	0.001808	3.81E-02	1.09E-03	1.71E-02
3768650	STAM2	11993811	FGF20	9.20E-10	4.53E-06	8.71E-05	2.52E-06	1.18E-01	3.25E-08	0.000115	1.25E-03	9.91E-05	8.50E-01
785915	GCNT1	11713331	PRICKLE2	1.30E-09	5.34E-05	5.01E-01	3.64E-05	3.37E-04	2.95E-07	3.82E-05	7.13E-05	4.26E-05	3.70E-02
785916	GCNT1	11713331	PRICKLE2	1.37E-09	6.08E-05	5.08E-01	4.21E-05	4.32E-04	5.51E-07	6.98E-05	1.31E-04	8.08E-05	6.61E-02
1202674	RPS6KA2	6061796	CDH4	2.46E-09	4.19E-05	3.78E-01	3.11E-05	1.12E-05	1.63E-08	0.000516	6.48E-03	3.18E-04	7.53E-04
1048471	ST3GAL1	2830096	APP	2.95E-09	2.43E-05	4.55E-04	1.84E-05	1.00E-01	9.63E-07	0.001105	1.11E-01	1.09E-03	3.65E-03
1025951	GALNT13	17568302	FMO2	3.40E-09	3.39E-05	3.16E-03	2.36E-05	2.26E-01	9.29E-07	0.002402	3.30E-03	1.94E-03	1.39E-02
6954	KIAA0467	4773873	ABCC4	3.67E-09	3.30E-06	1.24E-04	2.25E-06	3.11E-03	4.74E-07	0.003416	5.42E-01	1.59E-03	1.36E-02

To further evaluate the performance of the proposed statistic 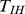, in Table 7 and Table S4 we list P-values for testing interaction calculated by the statistic 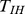, the “fast-epistasis” in PLINK and logistic regression using genotype coding. In Table 7 the 20 top pairs of interacting SNPs and in Table S4 the results of 233 pairs of interacting SNPs are presented. The P-values for interaction calculated by the statistic 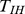 are much smaller than those from the “fast-epistasis” in PLINK and the logistic regression using genotype coding (Table 7 and Table S4). Moreover, the “fast-epistasis” in PLINK and the logistic regression coded by genotype detect very few interactions that can be replicated in two independent studies (Table 7 and Table S4). In fact, our results for all tested SNPs in 501 pathways showed that the “fast-epistasis” in PLINK and logistic regression coded by genotypes detected very few interactions that can be replicated in two studies (data not shown).

**Table 8:** A total of 18 significantly interacting SNPs identified by Bonferroni Correction.

SNP1 (rs)	Gene 1	Chrom 1	Position 1	SNP2 (rs)	Gene 2	Chrom 2	Position 2	P-value	
								Dataset 1	Dataset 2
1052248	LST1/NCR3	6	31664560	3131636	MICB	6	31584073	5.56E-013	7.76E-010
1052248	LST1/NCR3	6	31664560	3132468	MICB	6	31583465	8.41E-013	8.96E-010
443198	NOTCH4	6	32298384	3131636	MICB	6	31584073	1.13E-011	3.98E-008
626072	LAMA1	18	6941189	6121989	LAMA5	20	60350108	1.11E-015	5.67E-007
626072	LAMA1	18	6941189	4925386	LAMA5	20	60354439	1.47E-013	9.81E-007
7113099	NCAM1	11	112409545	10025210	SCD5	4	83858485	2.05E-011	1.00E-005
802509	CNTNAP2	7	145603003	1462140	HPSE2	10	100355999	2.29E-011	1.96E-005
832504	PLXNC1	12	93197019	13222291	KDELR2	7	6483965	2.55E-012	2.19E-005
2227956	HSPA1L	6	31886251	3134929	NOTCH4	6	32300085	7.76E-009	2.36E-011
3129869	HLA-DRA	6	32513649	3177928	HLA-DRA	6	32520413	3.77E-008	5.65E-014
3177928	HLA-DRA	6	32520413	9269080	HLA-DRB4	6	32548947	1.96E-007	3.70E-011
3129882	HLA-DRA	6	32517508	3177928	HLA-DRA	6	32520413	6.96E-007	2.71E-014
2620452	CNTNAP2	7	146644926	16982241	FUT2	19	53894671	1.50E-006	3.48E-012
1479838	CNTNAP2	7	146638597	16982241	FUT2	19	53894671	1.85E-006	1.77E-012
3134929	NOTCH4	6	32300085	3177928	HLA-DRA	6	32520413	2.81E-006	<1.00E-17
2856993	TAP2	6	32899381	9269080	HLA-DRB4	6	32548947	8.99E-006	1.71E-013
6498575	MYH11	16	15795817	9364864	RPS6KA2	6	166984655	1.14E-005	1.83E-012
935672	PRKCE	2	45899463	2744600	ALDH5A1	6	24641411	2.09E-005	1.88E-011

Eighteen significantly interacting SNPs identified by Bonferroni correction were listed in Table 8. In dataset1, the total number of SNPs for testing interaction was 50,327. The P-values for declaring interaction between SNPs after Bonferroni correction was 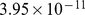. We found that there were 2,210 significant interactions with P-values less than 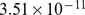 in the dataset 1. Then, interaction for all these 2,210 pairs of SNPs in the dataset 2 was examined. The P-values for declaring interaction between SNPs after Bonferroni correction in dataset 2 was 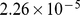. We identified eight significant interactions that were replicated in dataset 2. Similarly, if we started with dataset 2, the total number of SNPs for testing interaction was 49,876. The P-values for declaring interaction between SNPs after Bonferroni correction was 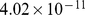. Significant interactions with the P-values less than 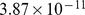 in dataset 2 were seen between 1,913 pairs of SNPs. Then, we tested for interaction for all these 1,913 pairs of SNPs in the dataset 1. The P-values for declaring interaction between SNPs after Bonferroni correction in dataset 1 was 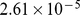, and 10 significant interactions were detected that were replicated in the dataset 1. A total of 9 interactions were common in Table 8 and Table S1 and Table S2.

## Discussion

The development of most diseases is a dynamic process of gene-gene and gene-environment interactions within a complex biological system. We expect that genome-wide interaction analysis will provide a possible source of finding missing heritability unexplained by current GWAS that test association individually. But, in practice, very few genome-wide interaction analyses have been conducted and few significant interaction results have been reported. Our aim is to develop statistical methods and computational algorithms for genome-wide interaction analysis which can be implemented in practice and provide evidence of gene-gene interaction. The purpose of this report is to address several issues to achieve this goal.

The first issue is how to define and measure interaction. Odds-ratio is a widely used measure of interaction for case-control design. The odds-ratio based measure of interaction between two loci is often defined as a departure from additive or multiplicative odds-ratios of both loci defined by genotypes. The genotype-based odds-ratio does not explore allelic association information between two loci generated by interaction between them in the cases. Any statistics that are based on genotype defined odds-ratio will often have low power to detect interaction. To overcome this limitation, we extended genotype definition of odds-ratio to haplotypes and revealed relationships between haplotype-defined odds-ratio and haplotype formulation of logistic regression. To further examine the validity of this concept, we studied the distribution of the test statistic under the null hypothesis of no interaction between two either linked or unlinked loci. Through extensive simulation (assuming allelic association in the controls), we show that the distribution of the haplotype odds-ratio-based statistic is close to a central 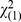 distribution even for small sample size and that type I error rates were close to the nominal significance levels.

The second issue is the power of the test statistic for genome-wide interaction analysis. The genome-wide interaction analysis requires testing billions of pairs of SNPs for interactions. The P-values for ensuring genome-wide significance level should be very small. Therefore, developing statistics with high power to detect interaction is an essential issue for the success of genome-wide interaction analysis. As an alternative to the logistic regression and the “fast-epistasis” in PLINK, we presented a haplotype odds-ratio-based statistic for detection of interaction between two loci and illustrated its power by extensive simulations. The power of the haplotype odds-ratio-based statistic ended up being a function of the measure of interaction and had much higher power to detect interaction than the “fast-epistasis” in PLINK and logistic regression.

The third issue is whether the interactions exist with no marginal association and how often they might occur in practice. Our data demonstrated that the majority of the significantly interacting SNPs showed no marginal association. Surprisingly, 75% of interacting SNPs with P-values (for testing marginal association) larger than 0.2 and 44% of interacting SNPs with P-values (for testing marginal association) larger than 0.5 in two studies were observed in our analysis. This strongly suggested that testing interaction for only SNPs with strong or mild marginal association will miss the majority of interactions.

The fourth issue is that of replication of the results. Genome-wide interaction analysis involves testing billions of pairs of SNPs. Even if after correction of multiple tests, the false positive results might be still high. To increase confidence in interaction test results, replication of interaction findings in independent studies is often sought. To date, very few results of genome-wide interaction analysis have been replicated. This begs the question whether the significant interaction can be replicated in independent studies. In this report, we show that interaction findings can be replicated in two independent studies.

The fifth issue is correction for multiple testing. Genome-wide interaction analysis often involves billions of tests, which would require an extremely small Bonferroni-corrected P-value to ensure a genome-wide significance level of 0.05. Replication of finding at such small P-values in independent studies is often extremely difficult. However, Bonferroni correction assumes that the tests are independent, yet many interaction tests are highly correlated. Correlations in the interaction tests come from two levels [23]. First, two pairs of SNPs may share a common SNP. Second, SNPs in the interaction tests may be dependent due to allelic association. The Bonferroni correction assuming independent tests will be overly conservative due to high correlations among the interaction tests. In this report, two strategies were used to tackle the multiple testing issues. The first is to use FDR to control type I error. The second is to replicate interaction finding. Replication allows us to detect the interactions that are frequent and consistent [22]. This approach still has the limitation that we still make independent assumption of the tests in calculation of FDR. Recently, Emily et al. (2009) [23] proposed to develop a Bonferroni-like correction for multiple tests based on the concept of the effective number of SNP pairs. The concept of the effective number of tests takes correlation among the tests into account and can be applied to both P-value and FDR correction [41]. This may be a promising approach to multiple test corrections in the genome-wide interaction analysis.

Although our data show that interactions can partially find the heritability of complex diseases missed by the current GWAS, they are still preliminary. Due to extremely intensive computations demanded by genome-wide interaction analysis we only tested interactions of a small set of SNPs which were located in the genes of 501 assembled pathways in a PC computer. The truly whole genome interaction analysis in which we will test for interactions between all possible pairs of SNPs across the genome has not been conducted. Gene-gene interaction is an important, though complex concept. The statistical interactions are scale dependent. There are a number of ways to define gene-gene interaction. How to define gene-gene interaction and develop efficient statistical methods and computational algorithms for genome-wide interaction analysis are still great challenges facing us. The main purpose of this report is to stimulate discussion about what are the optimal strategies for genome-wide interaction analysis. We expect that in coming years, genome-wide interaction analysis will be one of major tasks in searching for remaining heritability unexplained by the current GWAS approach.

## Supporting Information

Table S1 A total of 44 pairs of SNPs showing significant interaction with FDR less than 0.001 in two independent studies.
(0.04 MB XLS)

Table S2 A total of 211 pairs of interacted SNPs with FDR less than 0.003 in at two studies.
(0.07 MB XLS)

Table S3 P-values for testing association of single SNP and interaction between two SNPs.
(0.08 MB XLS)

Table S4 P-value for testing interaction calculated by T_IH_, PLINK and logistic regression using genotype coding.
(0.10 MB XLS)

Text S1 Appendices.
(0.06 MB DOC)
