# Supplementary material for: A Novel Statistic for Genome-Wide Interaction Analysis
Source: PLoS Genet. 2010 Sep 23;6(9):e1001131. doi: 10.1371/journal.pgen.1001131 (PMC2944798; doi:10.1371/journal.pgen.1001131)
Supplement: Text S1 — Appendices. (0.06 MB DOC) [file pgen.1001131.s005.doc]

**Text S1: Appendices**

**Appendix A**

Recall that

$\frac{OR_{G_{1}H_{1}}}{OR_{G_{1}H_{2}}OR_{G_{2}H_{1}}}=\frac{h_{11}h_{22}(1-h_{12})(1-h_{21})}{h_{12}h_{21}(1-h_{11})(1-h_{22})}.$ (A1)

But, we have

$\begin{matrix} \frac{h_{11}h_{22}}{h_{12}h_{21}}=\frac{P_{11}^{A}P_{22}^{A}/(P_{11}P_{22})}{P_{12}^{A}P_{21}^{A}/(P_{12}P_{21})} \\ \begin{matrix} & & =\frac{P_{11}^{A}P_{22}^{A}P_{12}P_{21}}{P_{12}^{A}P_{21}^{A}P_{11}P_{22}} \end{matrix}, \end{matrix}$ (A2)

and

$\begin{matrix} \frac{(1-h_{12})(1-h_{21})}{(1-h_{11})(1-h_{22})}=\frac{P_{12}^{N}P_{21}^{N}/(P_{12}P_{21})}{P_{11}^{N}P_{22}^{N}/(P_{11}P_{22})} \\ \begin{matrix} & & \begin{matrix} & & \begin{matrix} & =\frac{P_{12}^{N}P_{21}^{N}P_{11}P_{22}}{P{}_{11}^{N}P_{22}^{N}P_{12}P_{21}} \end{matrix} \end{matrix} \end{matrix}. \end{matrix}$ (A3)

Substituting equations (A2) and (A3) into equation (A1), we obtain

$\begin{matrix} \frac{OR_{G_{1}H_{1}}}{OR_{G_{1}H_{2}}OR_{G_{2}H_{1}}}=\frac{P_{11}^{A}P_{22}^{A}P_{12}P_{21}}{P_{12}^{A}P_{21}^{A}P_{11}P_{22}}\frac{P_{12}^{N}P_{21}^{N}P_{11}P_{22}}{P_{11}^{N}P_{22}^{N}P_{12}P_{21}} \\ \begin{matrix} & & & \begin{matrix} & =\frac{P_{11}^{A}P_{22}^{A}}{P_{12}^{A}P_{21}^{A}}\frac{P_{12}^{N}P_{21}^{N}}{P_{11}^{N}P_{22}^{N}} \end{matrix} \end{matrix}. \end{matrix}$ (A4)

Taking logarithm on both sides of equation (A4) yields

$$I_{GH}^{H}=\log\frac{P_{11}^{A}P_{22}^{A}}{P_{12}^{A}P_{21}^{A}}-\log\frac{P_{11}^{N}P_{22}^{N}}{P_{12}^{N}P_{22}^{N}}.$$

This completes the proof of equation (4).

**Appendix B**

Below we explain why the proposed statistic is not equivalent to the “fast-epistasis” test in

PLINK. The odds ratio for PLINK is defined in terms of combinations of alleles, not

haplotypes. These combinations of alleles are collapsed from genotypes and hence do not contain haplotype information. If we use $A\cap B,A\cap b,a\cap B$ and $a\cap b$to represent four groups: a group of alleles A and B, a group of alleles A and b, a group of alleles a and B, and a group of alleles a and b, respectively. Then, the log odds ratio in the test statistic defined in PLINK would be

$$OR_{PLINK}=[\log\frac{P(A\cap B|D=1)P(a\cap b|D=1)}{P(a\cap B|D=1)P(A\cap b|D=1)}-\log\frac{P(A\cap B|D=0)P(a\cap b|D=0)}{P(a\cap B|D=0)P(A\cap b|D=0)}].$$

The fast-epistasis test statistic is based on collapsing genotypes into groups of alleles. Therefore, the test statistic does not use haplotype information or gamete disequilibrium information. However, our log odds ratio is defined in terms of haplotype or extended concept of haplotype when loci are unlinked:

$$OR_{GH}=\log\frac{P_{11}^{A}P_{22}^{A}}{P_{12}^{A}P_{21}^{A}}-\log\frac{P_{11}^{N}P_{22}^{N}}{P_{12}^{N}P_{21}^{N}}.$$

$\frac{P(A\cap B|D=1)P(a\cap b|D=1)}{P(a\cap B|D=1)P(A\cap b|D=1)}$ is different from $\frac{P_{11}^{A}P_{22}^{A}}{P_{12}^{A}P_{21}^{A}}$. We use an example to show its difference. The data that are from Table 2.10 in the book “Genetic data analysis” (Weir, BS; 1990, Sinauer Associates, Inc. Publishers, Sunderland, Massachusetts) are copied here as Table B1. The haplotype frequencies and the frequencies of the groups of alleles in PLINK representation were listed in Tables B2 and B3, respectively. The odds ratio in our representation is equal to 7.8420. However, the odds ratio in PLINK representation is equal to 2.0370. This shows that the haplotype-based odds-ratio is different from the odds-ratio defined in PLINK. Therefore, the proposed statistic in this report is not equivalent to the “fast-epistasis” test in PLINK

Table B1. Data

|  | BB | Bb | bb |
| --- | --- | --- | --- |
| AA | 19 | 5 | 0 |
| Aa | 8 | 8 | 0 |
| aa | 0 | 0 | 0 |

Table B2. $2\times2$ Table in Haplotype frequencies.

|  | P_AB_=0.7262 | P_Ab_=0.0738 |
| --- | --- | --- |
|  | P_aB_=0.1113 | P_ab_=0.0887 |

Table B3. $2\times2$Table by PLINK representation.

|  | B | b |
| --- | --- | --- |
| A | 0.6875 | 0.1125 |
| a | 0.1500 | 0.050 |
